# Wellbeing of trans and gender‐diverse people with HIV in the United Kingdom: Results from Positive Voices 2022

**DOI:** 10.1111/hiv.70286

**Published:** 2026-08-27

**Authors:** H. Kitt, C. Kelly, A. Aghaizu, M. Ross, F. C. Lampe, C. J. Smith, V. Martin, C. Humphreys, A. Brown, A. Sparrowhawk, M. Kall, J. Sewell, E. Wainwright, V. Delpech, G. Davis, A. Farah, A. Fasanya, A. J. Rodger, T. Đuretić, PV2022 Study Group, Ann Sullivan, Rachel Jones, Mohammed Hassan, Serge Miodragovic, Ann Sullivan, Victoria Tittle, Mohammed Hassan, Serge Miodragovic, Sarah Edwards, Melanie Rosenvinge, Allison Mascagni, Rosa Harrington, Claudia Adade, Emily Clarke, Sandra Mason, Emily Clarke, Elaine Priest, Emily Clarke, Sandra Mason, Emily Clarke, Elaine Priest, Emily Clarke, Melissa Martin, Athavan Umaipalan, Julie Field, Clare Woodward, Felicity Williams, Liz Hamlyn, Lucy Campbell, Steve Taylor, Gerry Gilleran, Satwant Kaur, Cathy Ormiston, Laura Wilson‐Powell, Kate Saunders, Meena Gupta, Julia Ball, Mark Gompels, Louise Jennings, Malgorzata Slowinska, Angela Bailey, Sandra Rushwaya, Sarah Schoeman, Tadas Mazeika, Shalini Andrews, Laura Noonan, Liz Hamlyn, Lucy Campbell, Darren Cousins, Catherine Oliver, Daniel Clutterbuck, Connor Dalby, Amy Shepherd, Anura Piyadigamage, John Martin, Judith Zhou, Barbara Hayman, Emma Rutland, Paola Cicconi, Charlie Wells, Fiona Wilson, Claudia Krause, Su Jenkins, Anitha Vidhyadharan, Samantha Harwood, Lisa Goodall, Alison Bridgwood, Laura Wilson‐Powell, Suzanne Francis, Kirsty Mynard, Mandy Austin, Judith Zhou, Farai Mukazi, Chloe Hoskins, Ian Cormack, Jonathan Shaw, Amanda Smith, Nadia Khatib, Julie Walsh, Sarah Schoeman, Tadas Mazeika, Anitha Vidhyadharan, Brenda Hollier, Martin Jones, Penny Boxall, Ade Apoola, Catherine Gatford, John Evans‐Jones, Jennifer Harrison, Nashaba Matin, Moses Shongwe, Nashaba Matin, Moses Shongwe, Ian Fairley, Martin Jones, Zoe Cuthbertson, Penny Boxall, Chitra Babu, Denise Donahue, Fiona Burns, Katie Spears, Thomas Fernandez, Graham McKinnon, Rachael Bridgman, Ann Sullivan, James Hardie, Mohammed Hassan, Serge Miodragovic, Amanda Clarke, Lisa Barbour, Carole Cable, Mohanarathi Kawsar, Memory Kakowa, Richard Gilson, Gosala Gopalakrishnan, Abigail Severn, Anitha Vidhyadharan, Kate Castro‐Sanchez, Andrew Ustainowski, Fahd Niaz, Sophie Herbert, Helen Reboul, Ashini Fox, Sarah Chadwick, Nelson David, Megan Khan, Andrea Ng, Julia Rogers, Katie Saunders, Andrea Ng, Julia Rogers, Katie Saunders, Cathy Ormiston, Amandeep Gill, Laura Wilson‐Powell, Katie Saunders, Alison Blume, Natalie Parker, Jonathan Ross, Sindiso Masuka, Olubanke Davies, Analyn Alipustain, Maheshraj Radhakrishnan, Nadi Gupta, Nicola Williams, Helen Iveson, Ian Fairley, Angela Bailey, Sandra Rushwaya, Emma Street, Andrew Sealy, Ian Fairley, Tom Yucebiyik, Elbushra Herieka, Kevin Turner, Alison Blume, Felicity Young, Karen Rogstad, Jessica Mcneill, Gareth Stephens, Zoe Warwick, Angela Robinson, Elaine Freeman, Nashaba Matin, Moses Shongwe, Laura Hilton, Donna Stookes, Sarah Schoeman, Tadas Mazeika, Elizabeth Okecha, Nicola Fearnley, Jackie Todd, Sue Kimachia, Ann Sullivan, Sarah Edwards, Mohammed Hassan, Sophie Herbert, Helen Reboul, Jessica Daniel, Mary‐Jane Harding, Jonathan Shaw, Abbey Eboigbe, Ashley Hanson, Liz Hamlyn, Katie Toler, Fabian Chen, Emma Wainwright, Felix Kpodo, Nisha Pal, Clare Megson, Anitha Vidhyadharan, Matt Waller, David Chadwick, Jessica Roberts, Iain Reeves, Tracey Fong, Raouf Moussa, Melissa Milsom, Emma Street, Mike Ward, Lucy Twigger, Charlotte Swift, Raj Patel, Jane Whitehead, Sathish Thomas William, Jane Holder, Stephen Kegg, Rosa Harrington, Allison Mascagni, Claudia Adade, Lewis Haddow, Jessica Osorio, Ann Sullivan, Marie‐Louise Svensson, Sandra Underwood, Helen Pollitt, Anitha Vidhyadharan, Brenda Hollier, Ann Sullivan, Samantha Hill, Sara Scofield, Jenny Murira, Nicola Mackie, Sophia Taylor, Romina Tajik, Orla McQuillan, Denise Donahue, Judith Zhou, Rebecca Murdock, Elaine Banks

**Affiliations:** ^1^ UK Health Security Agency London UK; ^2^ Wessex School of Public Health Southampton UK; ^3^ CliniQ London UK; ^4^ Institute for Global Health, University College London London UK; ^5^ George House Trust Manchester UK; ^6^ Royal Berkshire NHS Foundation Trust Reading UK; ^7^ New South Wales Health Coffs Harbour Australia; ^8^ Royal Free London NHS Foundation Trust London UK

**Keywords:** anxiety, depression, gender diverse, gender identity, mental health, minority stress, non‐binary, quality of life, social support, suicide, transgender persons, transphobia

## Abstract

**Objectives:**

This national cross‐sectional observational study aimed to assess mental health, quality of life, supportive network, experiences of HIV‐related stigma in healthcare and unmet service need among trans and gender‐diverse people with HIV, compared to cisgender people with HIV.

**Methods:**

Positive Voices 2022 is a questionnaire survey of people accessing HIV care in England, Scotland and Wales carried out in 2022 and 2023 that included measures of depression and anxiety symptoms and suicidal ideation (PHQ‐9 and GAD‐7), health‐related quality of life (EQ‐5D‐5L), supportive network (modified Duke FSSQ), experiences of HIV‐related stigma in healthcare and service needs. Logistic and ordinal logistic regression assessed the association of gender identity with these measures unadjusted and adjusted for age group and ethnicity.

**Results:**

Overall, 67 trans or gender‐diverse and 4551 cisgender people with HIV participated. Compared to cisgender participants, trans and gender‐diverse participants were younger, more likely to be Asian and less likely to be black. They were less likely to be in employment, homeowners and financially secure. Trans and gender‐diverse participants had a higher prevalence of depression symptoms (35.5% [22/62] vs. 20.5% [865/4219], adjusted odds ratio [aOR] [95% confidence interval, CI] = 1.96 [1.15, 3.34]; *p* = 0.014) and suicidal ideation (37.1% [23/62] vs. 14.6% [616/4219]; aOR = 3.09 [1.85, 5.17]; *p* < 0.001) compared to cisgender participants. They also had poorer health‐related quality of life for three domains (anxiety and depression, usual activities and self‐care) and lower levels of supportive network in unadjusted and adjusted analyses. A higher proportion of trans and gender‐diverse participants than cisgender participants had unmet needs for health services, including mental health services (49.2% [31/63] vs. 30.2% [1310/4343]; aOR = 2.02 [1.22–3.35]; *p* = 0.007), lifestyle services, and welfare and social services.

**Conclusions:**

Trans and gender‐diverse people with HIV have poorer mental health and wellbeing than cisgender people with HIV, and a higher prevalence of unmet service needs, highlighting the need for access to culturally competent mental health and other health and welfare services.

## INTRODUCTION

Trans people have a gender identity that differs from their sex assigned at birth, whilst cis people's gender identity corresponds with their sex assigned at birth [1]. Gender diversity is an umbrella term encompassing trans, non‐binary and other people who identify beyond the binary categories of man or woman [2].

Trans and gender‐diverse people have an elevated prevalence of HIV [3], likely due to a complex interplay of social and systemic factors. Minority stress, stigmatization, marginalization, discrimination and intersectional forms of oppression, including transphobia and homophobia, may reduce access to health care, education and employment, and negatively impact mental and physical health and health‐promoting behaviours [4, 5, 6, 7]. Among trans and gender‐diverse people diagnosed with HIV, intersecting stigmas and discrimination may adversely impact access to health services, mental health and quality of life [8, 9].

There is a paucity of peer‐reviewed information regarding the health and well‐being of trans people with HIV [10]. Positive Voices is a recurring cross‐sectional survey of people accessing HIV care in England, Scotland and Wales. Positive Voices 2017 included 37 trans and gender‐diverse participants who were more likely to report being moderately to extremely anxious or depressed than cisgender participants (41% vs. 24%), and twice as likely to report having been refused or received delayed healthcare (23% vs. 11%) [10]. Using data from Positive Voices 2022 (PV2022), we explored differences by gender identity in the prevalence of depression and anxiety symptoms, suicidal ideation, quality of life, level of supportive network, experiences of HIV‐related stigma in healthcare and unmet service needs among people with HIV in the United Kingdom. Study participants included a significant proportion of all trans and gender‐diverse people with HIV in the United Kingdom and thus can add to our knowledge of the health and well‐being of this population.

## MATERIALS AND METHODS

### Study design, setting and participants

PV2022 was a cross‐sectional questionnaire survey of people accessing HIV care at 101 specialist HIV clinics in England, Scotland and Wales, carried out between April 2022 and March 2023 (methods provided in [11, 12]). People with HIV were randomly selected for inclusion on a recruitment list from each participating clinic using the national HIV and AIDS Reporting System (HARS) [13]. To maximize representation of trans and gender‐diverse people, all clinic attendees with these recorded gender identities were also added to the list. These individuals were then invited to participate by clinic staff. From December 2022, 14 clinics in London switched to sequential recruitment to increase participation.

Participants self‐completed a confidential questionnaire in either online or paper format; Topics asked about included socio‐demographics, HIV‐related and lifestyle factors, physical and mental health and well‐being, HIV‐related stigma and health service needs and usage.

The study was approved by the London Harrow Research Ethics Committee (13/LO/0279).

### Assessment of gender identity

Participants were asked ‘How do you identify your gender?’ with response options: Female (including trans woman); Male (including trans man); Non‐binary; In another way; Prefer not to say. Those who selected ‘non‐binary’ or ‘in another way’ were included in the trans and gender‐diverse category. Those that indicated in a subsequent question that their gender identity was not the same as their gender assigned at birth were also included in this category. All other participants were included in the cisgender category.

### Mental health and health‐related quality of life

Current depression symptoms were defined by a total score ≥10 on the PHQ‐9 [14]; current anxiety symptoms were defined by a total score ≥10 on the GAD‐7 [15]. PHQ‐9 and GAD‐7 assess symptoms over the past 2 weeks. Suicidal ideation was assessed using the ninth PHQ‐9 symptom (frequency of ‘thoughts that you would be better off dead or of hurting yourself in some way’ in the past 2 weeks; Figure 1d). Health‐related quality of life was assessed using the five EQ‐5D‐5L (EuroQol 5‐Dimension 5‐Level) domains: mobility; self‐care; pain and discomfort; usual activities; anxiety and depression, each with five response options ranging from no problems to extreme problems [16] (Figure 1b). EQ‐5D‐5L refers to how the participant felt on the day.

### Supportive network

Supportive network was assessed using six items from the Duke‐UNC Functional Social Support Questionnaire (FSSQ) [17]: ‘I have people who care what happens to me’, ‘I get love and affection’, ‘I get chances to talk to someone I trust about my personal or family problems’, ‘I get invitations to do things with other people’, ‘I get useful advice about important things in life’, and ‘I get help when I am sick in bed’, with five response options ranging from ‘as much as I would like’ (coded as 5) to ‘much less than I would like’ (coded as 1). A mean score across the six items was calculated and classified into five levels (Figure 1c).

### HIV‐related stigma in healthcare settings

Participants were asked ‘Because of your HIV status, have you ever experienced any of the following in your life’ followed by six statments relating to anticipated or enacted HIV‐related stigma in healthcare described below:

- *Anticipated HIV‐related stigma*: ‘been worried that you would be treated differently to other patients by healthcare staff’, ‘felt afraid to go to healthcare services because someone may learn your HIV status’ and ‘avoided going to healthcare services when you needed to’.
- *Enacted HIV‐related stigma*: ‘felt that you were not treated well in a healthcare setting’, ‘felt that you were refused healthcare or delayed treatment or a medical procedure’ and ‘heard healthcare staff gossiping about you (talking about you)’.

For each of the above, a binary variable was created combining answers of ‘yes, in the last year’ and ‘yes, but not in the last year’ compared to ‘no’, ‘don't know’, ‘prefer not to say’ and missing answers.

### Met and unmet service needs

Participants were asked about 29 services, which were grouped into five categories: (a) Mental health services (psychologist or counsellor; help to manage stress; help or advice regarding your sex life; help dealing with loneliness or isolation; relationship advice), (b) Lifestyle services (help to manage weight; help to stop smoking; alcohol counselling or treatment; drug counselling or treatment; chemsex support; drug detox or maintenance treatment), (c) HIV services (long term condition management support; peer support/social contact with other people with HIV; help sharing your HIV status; help managing side effects of HIV medications; information about living with HIV; HIV treatment advice; professional help to take your HIV tablets on time or correctly), (d) Welfare and social services (housing support; meal or food services; childcare services; employment advice and career skills; help claiming benefits; financial advice; legal advice; immigration support; domestic violence services) and (e) Other health services (family planning; home health services).

For each service, participants could select (1) ‘I have received this’, (2) ‘I needed this but could not get it’, (3) ‘I needed this but did not try to get it’, (4) ‘I needed this but did not know about it’ or (5) ‘I did not need this’. Answer options 2–4 represented an unmet service need, option 1 a met service need, and option 5 and missing answers represented no need.

### Other factors

Definitions and categorizations for other demographic, socio‐economic, lifestyle, HIV‐related and health‐related factors are given in Table 1.

### Statistical analyses

Participant characteristics were summarized descriptively, stratified by gender identity. The association of gender identity with depressive symptoms, anxiety symptoms, unmet service needs and HIV‐related stigma was assessed using binary logistic regression. Associations with each domain of health‐related quality of life (each with 5 response levels), with supportive network (5 levels) and with frequency of suicidal ideation (4 levels) were assessed using ordinal logistic regression. Outcome responses were coded such that a higher score corresponded to poorer health, lower support and more frequent suicidal ideation. Multivariable analyses were adjusted for age group (18–34, 35–44, 45–54, 55–64 and 65+ years) and ethnicity (White, black, Asian and other/missing). Further analyses were performed additionally adjusted for year of HIV diagnosis (1995 and earlier –2001, 2002–2013, 2014–2023, missing) and financial hardship (answer to the question ‘Do you have enough money for basic needs?’; ‘yes always’; ‘most of the time’; ‘some of the time’; ‘no’; missing) to assess whether these factors contributed to differences by gender identity in the outcome measures. Handling of missing values is described in the footnotes of figures and tables.

Analyses were conducted using Stata 18.0 (StataCorp, College Station, TX, USA).

## RESULTS

### Study population

Of 4622 people participating in PV2022, four provided insufficient demographic information and were excluded. Of the remaining 4618 participants, 67 (1.5%) identified as trans or gender diverse. Specifically, 27 were transgender women, 3 transgender men, 26 non‐binary people, 8 identified their gender in another way and 3 reported their gender to be different from their sex assigned at birth but preferred not to share their gender identity (Table 1). The remaining 4551 participants were categorized as cisgender (3423 cis men, 1097 cis women, 31 preferred not to say).

The trans and gender‐diverse participants were younger on average than cisgender participants (median = 46 years [interquartile range, IQR 35–57] vs. 53 [44–60]), more likely to be Asian (16% [11/67] vs. 4% [178/4551]) and less likely to be Black African (10% [7/67] vs. 21% [976/4551]). They were less likely to be in employment (49% [33/67] vs. 60% [2748/4551]), be homeowners (22% [15/67] vs. 41% [1867/4551]) or always have enough money for basic needs (33% [22/67] vs. 49% [2227/4551]). They were also more likely to be current or previous smokers and to report recent recreational drug use (Table 1).

**FIGURE 1:**
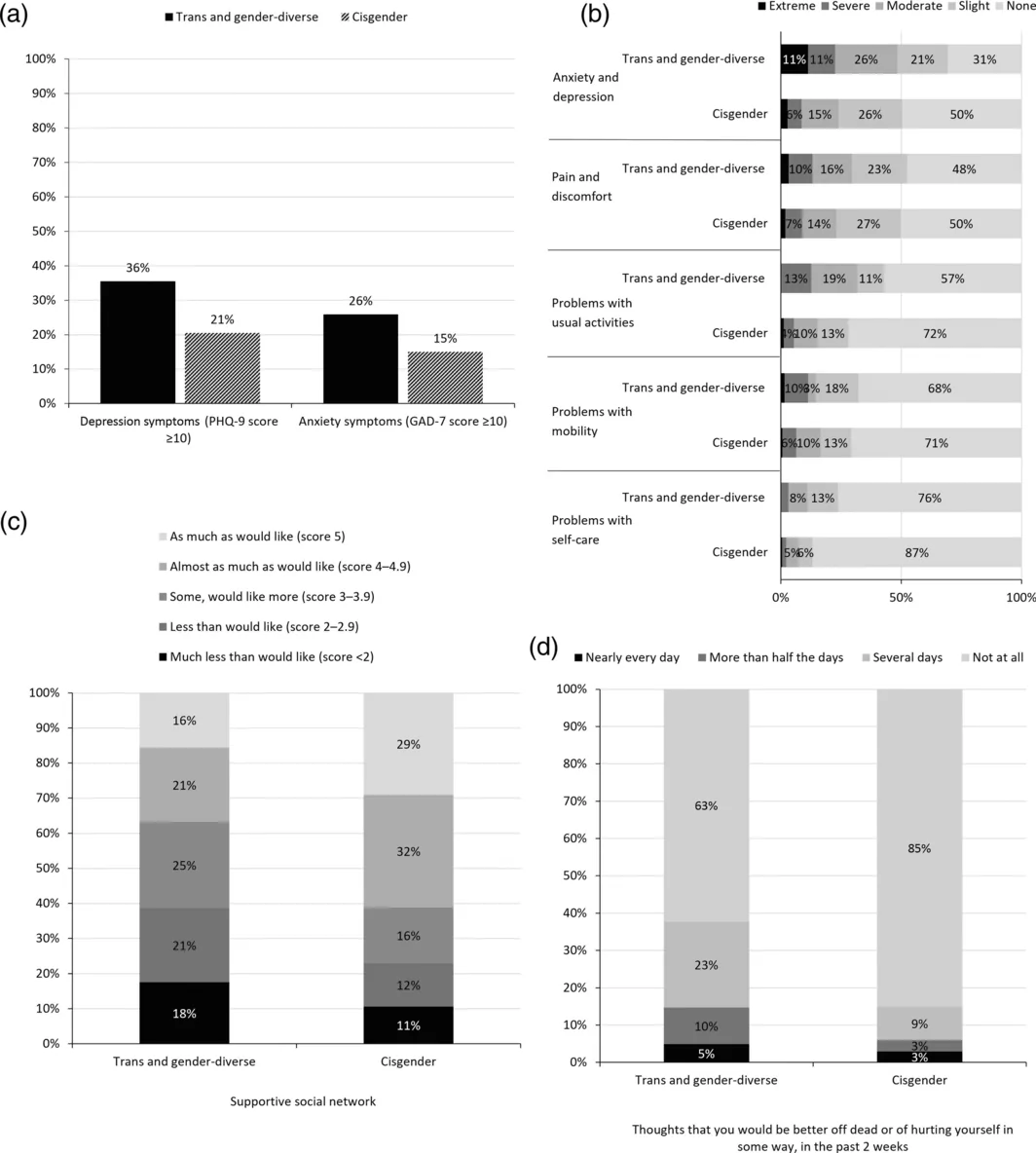
(a–d) Prevalence of depression symptoms, anxiety symptoms, poor quality of life, low supportive network, suicidal ideation by gender identity. (a) Symptoms of depression and anxiety: *N* = 62 trans and gender‐diverse and *N* = 4219 cisgender participants with data on depression symptoms; *N* = 58 trans and gender‐diverse and *N* = 4205 cisgender participants with data on anxiety symptoms. Participants who did not answer at least one question on PHQ‐9 (GAD‐7) were excluded from depression (anxiety) analyses; for the remaining participants, missing for an individual symptom was coded as absence of that symptom. GAD‐7, General Anxiety Disorder‐7; PHQ‐9, Patient Health Questionnaire‐9. (b) Health‐related quality of life (EQ‐5D‐5L domains): denominators for trans and gender‐diverse participants and cisgender participants, respectively, 62 and 4280 for anxiety and depression; 61 and 4280 for pain/discomfort; 63 and 4282 for usual activities; 62 and 4275 for mobility; 63 and 4281 for self‐care. (c) Supportive network: *N* = 57 trans and gender‐diverse and 4147 cisgender participants with data on supportive network. Participants who did not answer at least one question on modified Duke FSSQ were excluded from analysis; the average score across non‐missing items was used to impute missing items for individuals with 1–5 items missing. (d) Suicidal ideation (‘Thoughts that you would be better off dead or hurting yourself’): *N* = 62 trans and gender‐diverse and *N* = 4219 cisgender participants. Participants who did not answer at least one question on PHQ‐9 were excluded from suicidal ideation analysis; for the remaining participants, missing for the suicidal ideation question was coded as absence of that symptom.

**TABLE 1:** Characteristics of people with HIV who participated in PV2022, by gender identity (*N* = 4618).

Characteristics	Transgender and gender‐diverse		Cisgender	
	67		4551	
	*n* (column %)		*n* (column %)	
Age group (years)				
18–34	14	20.9%	355	7.8%
35–44	18	26.9%	867	19.1%
45–54	14	20.9%	1361	29.9%
55–64	17	25.4%	1380	30.3%
65+	4	6.0%	588	12.9%
Combined demographic group				
GBMSM	1	1.5%	2699	59.3%
Black African heterosexual men	0	0%	282	6.2%
Other heterosexual men	2	3.0%	374	8.2%
Men with unknown sexuality	0	0%	68	1.5%
Black African Women	1	1.5%	624	13.7%
Other Women	26	38.8%	473	10.4%
Non‐binary, in another way	34	50.7%	0	0%
Prefer not to say gender^a^	3	4.5%	31	0.7%
Ethnicity				
White (including white ethnic minorities)	38	56.7%	2951	64.8%
Black African	7	10.4%	976	21.4%
Black other	3	4.5%	135	3.0%
Asian	11	16.4%	178	3.9%
Mixed	3	4.5%	140	3.1%
Other/missing	5	7.5%	171	3.8%
Gender identity				
Man	3	4.5%	3423	75.2%
Woman	27	40.3%	1097	24.1%
Non‐binary	26	38.8%	0	0%
In another way	8	11.9%	0	0%
Prefer not to say	3	4.5%	31	0.7%
Sexuality				
Straight/heterosexual	21	31.3%	1573	34.6%
Gay or lesbian/homosexual	24	35.8%	2536	55.7%
Bisexual	3	4.5%	192	4.2%
Other	6	9.0%	18	0.4%
Prefer not to say/missing	13	19.4%	232	5.1%
Highest education level				
ISCED 1/2: primary or less	4	6.0%	160	3.5%
ISCED 3: upper secondary education	17	25.4%	1470	32.3%
ISCED 4/5: technical and vocational	6	9.0%	454	10.0%
ISCED 6: bachelors or equivalent level	19	28.4%	930	20.4%
ISCED 7/8: postgraduate degree	10	14.9%	987	21.7%
Other/missing	11	16.4%	550	12.1%
Employment status				
Employed	33	49.3%	2748	60.4%
Unemployed	8	11.9%	310	6.8%
Retired	5	7.5%	572	12.6%
Not working due to sickness or disability	10	14.9%	364	8.0%
Other, including student or carer/missing	11	16.4%	557	12.2%
Money for basic needs (food, rent, bills, etc.)				
Yes, always	22	32.8%	2227	48.9%
Most of the time	24	35.8%	1070	23.5%
Some of the time	9	13.4%	539	11.8%
No	3	4.5%	283	6.2%
Missing	9	13.4%	432	9.5%
Housing status				
Homeowner	15	22.4%	1867	41.0%
Renting privately	17	25.4%	1018	22.4%
Renting from council or housing association	22	32.8%	947	20.8%
Temporary accommodation or homeless	0	0%	44	1.0%
Other (rent‐free, sheltered, care home)	4	6.0%	236	5.2%
Missing	9	13.4%	439	9.7%
Year of HIV diagnosis				
1995 and earlier	5	7.5%	474	10.4%
1996–2001	7	10.5%	651	14.3%
2002–2007	14	20.9%	1185	26.0%
2008–2013	17	25.4%	1164	25.6%
2014–2019	17	25.4%	897	19.7%
2020–2023	4	6.0%	93	2.0%
Missing	3	4.5%	87	1.9%
BMI (self‐reported) in kg/m^2^				
Underweight (BMI <18.5)	1	1.5%	77	1.7%
Normal weight (BMI 18.5–24.9)	22	32.8%	1493	32.8%
Overweight (BMI 25–29.9)	17	25.4%	1284	28.2%
Obese (BMI 30–39.9)	11	16.4%	902	19.8%
Missing	16	23.9%	795	17.5%
Smoking status				
Never smoker	18	26.9%	2048	45.0%
Previous smoker	24	35.8%	1304	28.7%
Current smoker	16	23.9%	697	15.3%
Prefer not to say/missing	9	13.4%	502	11.0%
Alcohol consumption frequency				
Never	14	20.9%	1017	22.4%
Monthly or less	18	26.9%	947	20.8%
2–4 times a month	12	17.9%	761	16.7%
2–3 times a week	12	17.9%	882	19.4%
≥4 times a week^b^	3	4.5%	512	11.3%
Missing	8	11.9%	432	9.5%
Recreational drug use in last 3 months				
No	35	52.2%	3073	67.5%
Yes	23	34.3%	976	21.4%
Missing	9	13.4%	502	11.0%
Diagnosed comorbidities^c^				
None/missing	50	74.6%	2883	63.3%
1	11	16.4%	1020	22.4%
2–3	5	7.5%	414	9.1%
4 or more	1	1.5%	234	5.1%

Abbreviations: BMI, body mass index; GBMSM, gay, bisexual and other men who have sex with men; ISCED, International Standard Classification of Education.

^a^ Three people in the trans and gender‐diverse group have an unknown gender (they selected ‘prefer not to say’ their gender, but did say that their gender was different to their sex assigned at birth).

^b^ Participants selected from a list of drugs they have used in the last 3 months which included (with street names also given): cannabis, ecstasy, crystal meth, gamma hydroxybutyrate (GHB)/gamma butyrolactone (GBL), amphetamine, amyl nitrate, cocaine, ketamine, mephedrone, acid or LSD, crack, heroin, anabolic steroids, other (specify). Viagra is not included.

^c^ Includes a previous diagnosis of: cardiovascular disease (heart attack, angina or coronary heart disease, stroke, TIA), diabetes, arthritis, rheumatoid arthritis, cancer, chronic obstructive pulmonary disease, kidney disease, liver disease.

### Mental health, health‐related quality of life and supportive network

Compared to cisgender participants, trans and gender‐diverse participants had a higher prevalence of depression symptoms (35.5% [22/62] vs. 20.5% [865/4219], *p* = 0.005) and anxiety symptoms (25.9% [15/58] vs. 15.0% [630/4205], *p* = 0.024; Figure 1a, Table 2). In analysis adjusted for age and ethnicity, trans and gender‐diverse participants had 96% increased odds of depression symptoms (adjusted odds ratio, aOR [95% confidence interval] = 1.96 [1.15, 3.34]; *p* = 0.014) compared to cisgender participants (Table 2). There was also some evidence of increased odds of anxiety symptoms (aOR [95% CI] = 1.75 [0.96, 3.21]; *p* = 0.068).

**TABLE 2:** Unadjusted and adjusted associations of gender identity with mental health, health‐related quality of life, supportive network, suicidal ideation and unmet service needs.

*N* = 4618^a^	Transgender and gender‐diverse participants compared to cisgender participants					
	Unadjusted odds ratio (95% CI)	*p*	Adjusted (1) odds ratio^b^ (95% CI)	*p*	Adjusted (2) odds ratio^c^ (95% CI)	*p*
Depression symptoms (PHQ‐9 ≥ 10)^d^, ^e^	2.13 (1.26–3.61)	0.005	1.96 (1.15–3.34)	0.014	1.69 (0.96–2.96)	0.069
Anxiety symptoms (GAD‐7 ≥ 10)^d^, ^e^	1.98 (1.09–3.58)	0.024	1.75 (0.96–3.21)	0.068	1.56 (0.83–2.92)	0.169
Lower supportive network^f^	2.24 (1.42–3.52)	0.001	2.25 (1.42–3.55)	0.001	2.00 (1.28–3.15)	0.003
Poor quality of life (EQ‐5D)^f^, ^g^						
Problems with mobility	1.14 (0.67–1.93)	0.621	1.51 (0.87–2.62)	0.141	1.37 (0.78–2.41)	0.259
Problems with self‐care	1.98 (1.11–3.53)	0.021	2.48 (1.37–4.50)	0.003	2.17 (1.16–4.04)	0.015
Problems with usual activities	2.12 (1.30–3.45)	0.002	2.48 (1.50–4.07)	<0.001	2.18 (1.31–3.64)	0.003
Pain and discomfort	1.23 (0.76–1.98)	0.402	1.50 (0.92–2.44)	0.103	1.32 (0.81–2.15)	0.262
Anxiety and depression	2.78 (1.75–4.43)	<0.001	2.48 (1.55–3.97)	<0.001	2.19 (1.37–3.51)	0.001
Thoughts that you would be better off dead or of hurting yourself in some way^d^	3.24 (1.96–5.35)	<0.001	3.09 (1.85–5.17)	<0.001	2.87 (1.70–4.84)	<0.001
Unmet service needs^e^						
Any unmet mental health service need	2.24 (1.36–3.69)	<0.001	2.02 (1.22–3.35)	0.007	1.83 (1.09–3.08)	0.023
Any unmet lifestyle service need	2.58 (1.57–4.26)	<0.001	2.42 (1.45–4.04)	0.001	2.28 (1.35–3.84)	0.002
Any unmet HIV service need	1.70 (1.02–2.85)	0.042	1.47 (0.87–2.49)	0.15	1.33 (0.78–2.27)	0.302
Any unmet welfare and social service need	2.22 (1.33–3.69)	0.002	2.12 (1.25–3.60)	0.005	1.78 (1.00–3.17)	0.049
Any unmet other health service need	2.73 (1.33–5.59)	0.006	2.54 (1.21–5.31)	0.014	2.12 (0.99–4.57)	0.054

Abbreviations: CI, confidence interval; EQ‐5D, EuroQol 5‐Dimension 5‐Level; GAD‐7, General Anxiety Disorder‐7; PHQ‐9, Patient Health Questionnaire‐9.

^a^ *N* = 4281 for depression symptoms; 4263 for anxiety symptoms; 4337 for mobility; 4344 for self‐care; 4345 for usual activities; 4341 for pain or discomfort; 4342 for anxiety or depression; 4204 for supportive network; 4281 for suicidal ideation; 4406 for unmet service needs.

^b^ Adjusted 1: adjusted for age group (18–34, 35–44, 45–54, 55–64, 65+ years) and ethnicity (white, black, Asian, other/missing).

^c^ Adjusted 2: adjusted for age group (18–34, 35–44, 45–54, 55–64, 65+ years), ethnicity (white, black, Asian, other/missing), year of HIV diagnosis (1995 and earlier–2001, 2002–2013, 2014–2023, missing) and enough money for basic needs (yes always, yes most of the time, yes some of the time, no, missing).

^d^ Answers refer to symptoms over a 2‐week period.

^e^ Logistic regression.

^f^ Ordinal logistic regression. Odds ratios represent the odds of being one level poorer for quality of life domains and supportive network and one level more frequent for suicidal ideation.

^g^ Answers refer to how the participant felt on the day.

Considering health‐related quality of life, a greater percentage of trans and gender‐diverse participants reported problems compared to cisgender participants across three of the five EQ‐5D‐5L domains: self‐care; usual activities; anxiety and depression (Figure 1b). Associations remained strong in analysis adjusted for age and ethnicity (Table 2).

Overall, 38.6% (22/57) of trans and gender‐diverse participants had less or much less social support than they would like (modified Duke FSSQ average score <3) versus 22.9% (951/4147) of cisgender participants. (Figure 1c). After adjustment for age and ethnicity, trans and gender‐diverse people had 125% increased odds of being in a poorer social support category (aOR [95% confidence interval, CI] = 2.25 [1.42, 3.55] vs. cisgender; *p* = 0.001).

Overall, 37.1% (23/62) of trans and gender‐diverse participants had thoughts of being better off dead or of hurting themselves during the previous 2 weeks, compared with 14.6% (616/4219) of cisgender participants, (Figure 1d) and this association remained in analysis adjusted for age and ethnicity (aOR [95% CI] of being in a more frequent suicidal ideation category = 3.09 [1.85‐5.17]; *p* < 0.001) (Table 2).

In analyses additionally adjusted for year of HIV diagnosis and financial hardship, associations were attenuated, but trans and gender‐diverse gender remained significantly associated with lack of supportive network, three quality of life domains (anxiety and depression, usual activities and self‐care) and suicidal ideation (Table 2).

### HIV‐related stigma in healthcare settings

The proportion of participants who thought that they had ever been refused healthcare or delayed treatment or a medical procedure because of their HIV status was higher for trans and gender‐diverse than cisgender participants (20.7% [12/58] vs. 10.1% [425/4204]) (Table 3). aOR [95% CI] = 2.34; [1.22, 4.48]; *p* = 0.010 after adjustment for age and ethnicity). This association remained when additionally adjusting for year of HIV diagnosis and financial hardship. There was also some evidence that trans and gender‐diverse people were more likely to feel they had not been treated well in a healthcare setting (24.1% [14/58] vs. 15.2% [638/4204], aOR [95% CI] = 1.76 [0.95–3.24] *p* = 0.072) after adjustment for age and ethnicity. There were no significant differences by gender identity for these other measures of HIV‐related stigma in health services, and the magnitude of differences was small.

**TABLE 3:** Prevalence of HIV‐related stigma in healthcare, unadjusted and adjusted associations of gender identity with experiences of HIV‐related stigma in healthcare.

*N* = 4618	Transgender and gender‐diverse participants compared to cisgender participants									
	Trans and gender‐diverse		Cisgender		Unadjusted odds ratio (95% CI)	*p*	Adjusted (1) odds ratio^a^ (95% CI)	*p*	Adjusted (2) odds ratio^b^ (95% CI)	*p*
	*N*	%	*N*	%						
Ever felt afraid to go to healthcare services because someone may learn your HIV status										
No	45	77.6%	3203	76.2%	0.92 (0.50–1.72)	0.804	0.84 (0.45–1.57)	0.584	0.81 (0.43–1.53)	0.525
Yes	13	22.4%	1001	23.8%						
Ever avoided going to healthcare services when you needed to because of your HIV status										
No	47	81.0%	3.658	87.0%	1.57 (0.81–3.04)	0.183	1.39 (0.71–2.72)	0.342	1.27 (0.64–2.51)	0.496
Yes	11	19.0%	546	13.0%						
Ever been worried that you would be treated differently to other patients by healthcare staff because of your HIV status										
No	40	69.0%	2985	71.0%	1.10 (0.63–1.93)	0.734	1.01 (0.57–1.78)	0.976	0.96 (0.54–1.71)	0.899
Yes	18	31.0%	1219	29.0%						
Ever heard healthcare staff gossiping about you (talking about you) because of your HIV status										
No	51	87.9%	3853	91.7%	1.51 (0.68–3.34)	0.314	1.60 (0.72–3.59)	0.251	1.50 (0.67–3.40)	0.327
Yes	7	12.1%	351	8.4%						
Ever felt that you were not treated well in a healthcare setting because of your HIV status										
No	44	75.9%	3566	84.8%	1.78 (0.97–3.26)	0.063	1.76 (0.95–3.24)	0.072	1.62 (0.87–3.03)	0.127
Yes	14	24.1%	638	15.2%						
Ever felt that you were refused healthcare or delayed treatment or a medical procedure because of your HIV status										
No	46	79.3%	3779	89.9%	2.32 (1.22–4.41)	0.01	2.34 (1.22–4.48)	0.010	2.25 (1.16–4.37)	0.017
Yes	12	20.7%	425	10.1%						

*Note*: *N* = 58 trans and gender‐diverse and *N* = 4204 cisgender participants. Participants who did not answer at least one stigma question were excluded from analysis; for the remaining participants, missing for any stigma question was coded as absence of that stigma.

Abbreviation: CI, confidence interval.

^a^ Adjusted 1: adjusted for age group (18–24, 35–44, 45–54, 55–64, 65+ years) and ethnicity (white, black, Asian, other/missing).

^b^ Adjusted 2: adjusted for age group (18–24, 35–44, 45–54, 55–64, 65+ years), ethnicity (white, black, Asian, other/missing), year of HIV diagnosis (1995 and earlier–2001, 2002–2013, 2014–2023, missing) and enough money for basic needs (yes always, yes most of the time, yes some of the time, no, missing).

### Service needs

For all five service types, the prevalence of need tended to be higher for trans and gender‐diverse participants versus cisgender participants (Figure 2a–e). Furthermore, trans and gender‐diverse participants had a higher prevalence of unmet need than cisgender participants for all service types: 49.2% [31/63] vs. 30.2% [1310/4343] for mental health, 46.0% [29/63] vs. 24.8% [1078/4343] for lifestyle, 38.1% [24/63] vs. 26.5% [1152/4343] for HIV, 39.7% [25/63] vs. 22.9% [993/4343] for welfare and social, 14.3% [9/63] vs. 5.8% [250/4343] for other health services (Figure 2a–e); Table 2. Associations with unmet need for mental health services, lifestyle services, welfare and social services and other health services remained significant after adjustment for age and ethnicity and, with the exception of other health services, after additional adjustment for years of HIV diagnosis and financial hardship (Table 2).

**FIGURE 2:**
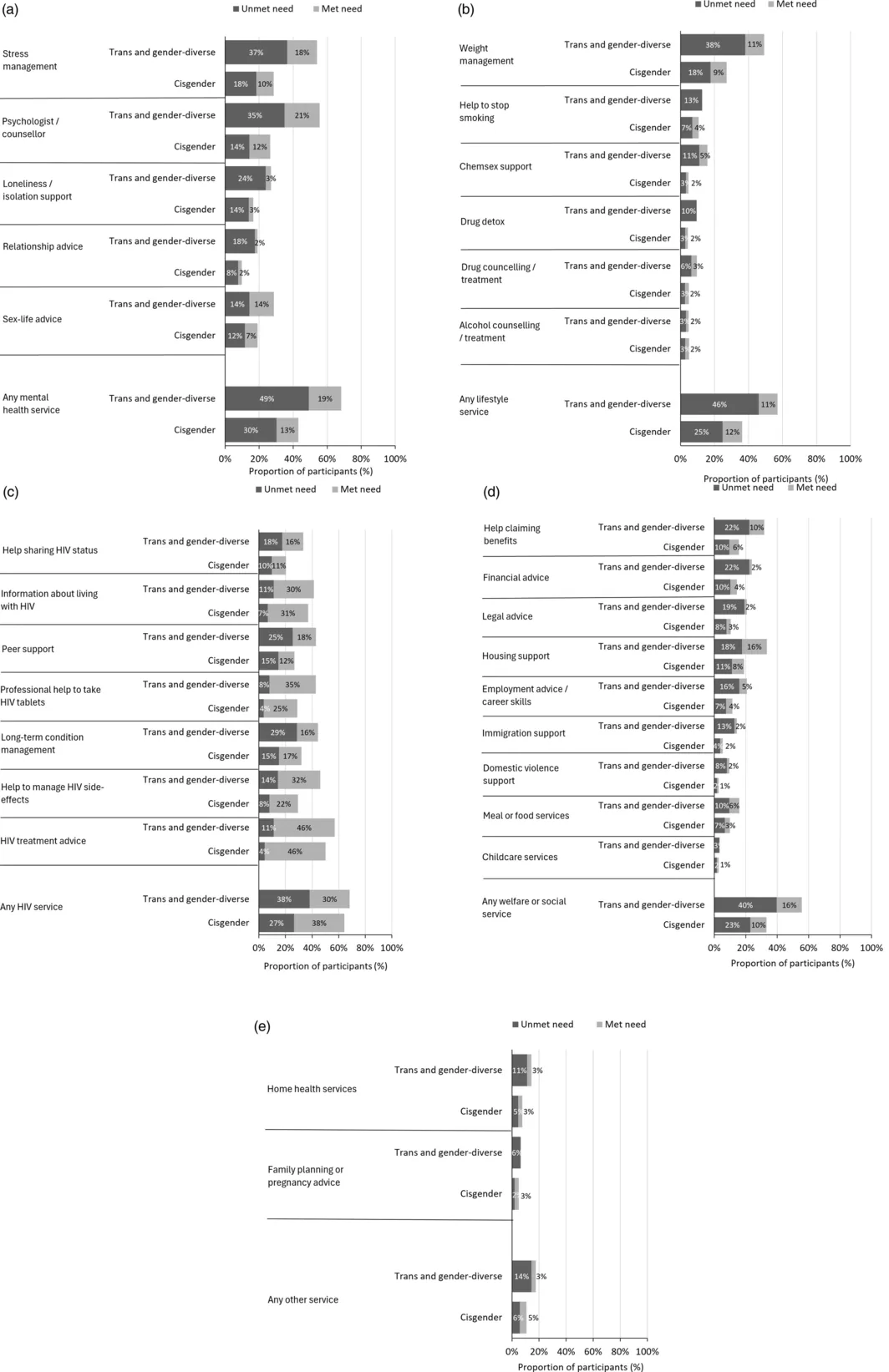
(a–e): Unmet and met service need, by gender identity. (a) Mental health services. (b) Lifestyle services. (c) HIV services. (d) Welfare and social services. (e) Other health services. For each of (a) to (e) *N* = 63 trans and gender‐diverse and *N* = 4343 cisgender participants. Participants who did not answer at least one of all above service need question were excluded from analysis; for the remaining participants, missing for a particular service need question was coded as absence of need for that service.

## DISCUSSION

This study provides insight into the wellbeing of trans and gender‐diverse people with HIV in the UK: they experienced a higher prevalence of depression symptoms, suicidal ideation, poor health‐related quality of life in specific domains, and lower levels of supportive network than cisgender people with HIV after accounting for differences in age and ethnicity. There was also some evidence of a higher prevalence of anxiety symptoms. They also reported a higher prevalence of unmet need for services including mental health, lifestyle and welfare and social services, in comparison to cisgender people with HIV.

These findings align with a recent systematic review of 63 global studies which highlighted a high burden of mental health challenges (40% depressive symptoms, 29% suicidal ideation and 39% anxiety symptoms) among transfeminine people living with HIV [18].

Another meta‐analysis of 41 studies found that minority stress among people identifying with a sexual or gender minority group is a risk factor for non‐suicidal self‐injury [19], and in an online Hungarian survey of 202 trans and gender‐diverse adults, trans men, gender‐diverse people and younger trans or gender‐diverse people had a particularly high risk of non‐suicidal self‐injury [20]. Despite this, there remain few peer‐reviewed comparative studies considering the prevalence of poor mental health compared to cisgendered people with HIV. Our findings provide evidence that there is a greater mental health burden among trans and gender‐diverse people with HIV, which should be studied further.

Other factors which may influence mental health among people with HIV include years living with HIV [21] and social vulnerability (including socio‐economic hardship, poor living situation and lack of social support) [22]. The higher prevalence of mental health symptoms among trans and gender‐diverse people with HIV may be due to an interplay of social and societal factors including intersecting stigmas, minority stress and marginalization, as well as higher levels of socioeconomic hardship. In our study, the higher prevalence of mental health problems and poor wellbeing for trans and gender‐diverse participants compared to cisgender participants was attenuated somewhat after further adjustment for years since HIV diagnosis and financial hardship, suggesting that a part of the difference is due to, and may be mediated by, greater socio‐economic disadvantage among the trans and gender‐diverse participants.

Long wait times for gender‐affirming care may also exacerbate mental health conditions [23]. A cohort study of 104 transgender and non‐binary youths aged 13–20 found 60% lower odds of depression symptoms (PHQ‐9), and 73% lower odds of self‐harm or suicidal thoughts, associated with receipt of gender‐affirming interventions [24]. Information on access to gender‐affirming care was not collected in PV2022. However, reports indicate that there are long wait times for access to initial assessment at clinics providing gender‐affirming care in the United Kingdom of almost 7 or 8 years [25, 26].

Our study suggests that trans and gender‐diverse people with HIV were much less likely than cisgender people with HIV to have a supportive social network, which may exacerbate minority stress and isolation and has previously been linked to the onset and relapse of depression [27]. Interventions to promote social cohesion have been suggested to improve mental health [28, 29], including the World Professional Association for Transgender Health (WPATH) Standards of Care for the Health of Transgender and Gender‐Diverse People recommendation that mental health professionals encourage, support and empower transgender and gender‐diverse people to develop and maintain social support systems [2].

Irrespective of gender identity, a substantial proportion of PV2022 participants reported at least one of the six types of anticipated or experienced HIV‐related stigmas in healthcare settings (overall 20% of participants in the past year and a further 22% not in the last year). Being refused healthcare or having delayed treatment or a medical procedure because of their HIV status was the only HIV‐related stigma in healthcare measure that was significantly associated with identifying as trans or gender‐diverse, being reported by twice as many participants in this group. This may give evidence of intersecting discriminations related to gender identity and HIV, and/or may relate to delay in receipt of gender services. Our measures specifically referred to HIV‐related stigma in healthcare and therefore did not capture additional stigma related to gender identity or other intersectional factors such as ethnicity.

The national Lesbian, Gay, Bisexual and Transgender (LGBT) survey launched by the Government Equalities Office in July 2017 received 108 100 responses from adults living in the United Kingdom, 13% of whom were trans or non‐binary [30]. Here, 40% of trans respondents who had accessed or tried to access NHS healthcare services reported experiencing at least one negative experience because of their gender identity in the previous year. Additionally, 21% reported that their specific needs were ignored or not taken into account when accessing healthcare services in the last year, and 18% had avoided subsequent health care treatment for fear of a negative reaction [30].

In PV2022, some of the highest proportions of unmet needs among the trans and gender‐diverse participants were for mental health services, specifically 37% had an unmet need for stress management, 35% for access to a psychologist or counsellor, 24% for loneliness/isolation support. A higher proportion of trans and gender‐diverse participants compared to cisgender participants reported unmet needs across a range of different services in addition to mental health. Prevalence of unment need among trans and gender‐diverse participants was also particularly high for help claiming benefits (22%), financial advice (22%) and weight management (38%), and in terms of HIV services, peer support (25%) and long‐term condition management (29%). However for HIV services, levels of met need also tended to be high.

Mental health service needs are not unique to people with HIV. There was a 44% rise in referrals to NHS mental health services between 2016–2017 and 2021–2022 [31], and a further 15% increase between 2022–2023 and 2024–2025 [32]. This increased demand has been coupled with long wait lists [33, 34]. People with HIV, including trans and gender‐diverse people could benefit from any improvements in waiting times implemented for the UK general population.

As seen in the current study among people with HIV, results from the national LGBT survey highlight that unmet need for mental health services disproportionately affects trans and gender‐diverse people: 14% had an unmet need for mental health services compared to 7% of cisgender participants [30]. Additionally, 28% of trans and gender‐diverse respondents who had accessed or tried to access mental health services in the previous year did not find it easy to do so, most commonly due to long waiting lists and unsupportive GPs, and only half (51%) of trans people had a positive experience [30].

PV2022 findings show that increased mental health support is needed for people with HIV, regardless of gender identity. However, it also shows that unmet needs for mental health services and across a range of other health service types disproportionately affect trans and gender‐diverse people with HIV. This suggests that we should integrate provision of pyschosocial support services within HIV clinics and gender‐affirming care clinics.

### Limitations

Although PV2022 is broadly representative of all people living with HIV and accessing care in England in 2022, there remains an over‐representation of men (6.6% absolute difference), people of white ethnicity (13.5%) and aged 55–64 years (6.0%) [12, 13]. In addition, PV2022 is designed to elucidate the experiences of the population of people with HIV as whole, making this study a secondary analysis of the trans and gender‐diverse people with HIV within that wider population with questions relating to HIV and general health experiences, rather than to the trans and gender‐diverse experience specifically. Further, the study does not provide information on the trans general population. The cross‐sectional design precludes causal inferences, and there may be confounding or mediating factors not measured, including neurodivergence and access to gender‐affirming care. Recruiting through HIV clinics means that the study population is likely to disproportionately include those who are better engaged in care, have time to participate and can speak English. We are also only able to include those who felt able to disclose their gender identity in the trans and gender‐diverse group. In our analysis, trans and gender‐diverse people were grouped together due to small numbers, and confidence intervals for associations are relatively wide. We have referred to participants that accessed services as having a ‘met service need’, but they may have accessed a service without having their needs met. The ‘met service need’ may therefore be overestimated. When asking about need for mental health services, the categories were broad and did not include help for diagnoses of serious mental health disorders (e.g., schizophrenia).

## CONCLUSIONS

Our findings suggest that trans and gender‐diverse people with HIV are more likely to have poorer mental health and wellbeing than cisgender people with HIV. They also have lower levels of supportive networks, which can be a key factor in protecting against depression, anxiety and other adverse mental health outcomes. A greater proportion of trans and gender‐diverse people reported unmet needs for mental health, lifestyle and welfare and social services. This highlights the need to ensure people with HIV, including trans and gender‐diverse people, are assessed for poor psychological health and related factors, including loneliness, social isolation and socioeconomic needs. Further, timely receipt of psychosocial support through culturally competent services tailored to trans and gender‐diverse populations, possibly embedded into HIV care, in addition to gender‐affirming care and community‐led services, is crucial. Other approaches could include implementing anti‐discrimination initiatives targeting both gender‐diverse identity and HIV status and policies that address structural inequities. Further, a significant proportion of people with HIV experienced HIV‐related stigma in healthcare, highlighting continued need for interventions that address this.

## AUTHOR CONTRIBUTIONS

CK, AA, FCL, CJS, VM, CH, AB, AS, MK, JS, VD, AJR conceived, designed, planned and conducted the Positive Voices 2022 study; HK, AFar contributed to study co‐ordination and data collection; EW contributed to data collection; AS leads personal and public involvement for Positive Voices 2022; GD and AFas contributed to knowledge mobilisation. HK, FCL, CJS, AJR conceived and designed the analysis for this manuscript; HK, FCL, CJS conducted the statistical analysis; HK wrote the first draft of the manuscript; TD provided support for the lead author and oversaw the project; MR provided important insights from the community perspective; all authors provided critical comment and revision of the draft manuscript.

## FUNDING INFORMATION

This study represents independent research funded by the National Institute for Health Research (Programme Grants for Applied Research, A person‐centred Needs Informed model of Care for people with HIV, to improve well‐being, mental health and reduce socio‐economic disadvantages and stigma [NICHE]: NIHR202038). The views expressed in this publication are those of the authors and not necessarily those of the NHS, the National Institute for Health Research or the Department of Health and Social Care. Funding was also received from the Gilead Sciences Ltd.

## CONFLICT OF INTEREST STATEMENT

The authors declare no conflicts of interest.

## ETHICS STATEMENT

The study was approved by the London Harrow Research Ethics Committee (ref. 13/LO/0279).

## Supporting information


**Data S1:** Full list of clinic staff and sites.

## Data Availability

Research data are not shared.
