# Supplementary material for: Wellbeing of trans and gender‐diverse people with HIV in the United Kingdom: Results from Positive Voices 2022
Source: HIV Med. 2026 Aug 27;27(11):1763–77. doi: 10.1111/hiv.70286 (PMC13725706; doi:10.1111/hiv.70286)
Supplement: Supplementary file 1 [file HIV-27-1763-s001.docx]

**https://www.gov.uk/government/publications/hiv-positive-voices-survey/positive-voices-2022-survey-report-appendices#appendix-10-clinic-collaborators**

**Clinic collaborators**

- 10 Hammersmith Broadway Clinic, London (Ann Sullivan, Rachel Jones, Mohammed Hassan, Serge Miodragovic)
- 56 Dean Street, London (Ann Sullivan, Victoria Tittle, Mohammed Hassan, Serge Miodragovic)
- Abbey View Clinic, iCaSH Suffolk, Bury St Edmunds (Sarah Edwards)
- Alexis Clinic, Alexis Clinic, London (Melanie Rosenvinge, Allison Mascagni, Rosa Harrington, Claudia Adade)
- Axess Clinic, Bath Street Health and Wellbeing Centre, Warrington (Emily Clarke, Sandra Mason)
- Axess Clinic, Eagle Bridge Health and Well Being Centre, Crewe (Emily Clarke, Elaine Priest)
- Axess Clinic, Halton General Hospital (Emily Clarke, Sandra Mason)
- Axess Clinic, Macclesfield Hospital (Emily Clarke, Elaine Priest)
- Axess Clinic, Royal Liverpool University Hospital (Emily Clarke, Melissa Martin)
- Barking Community Hospital Sexual Health Clinic, London (Athavan Umaipalan, Julie Field)
- BBV Clinic, Milton Keynes University Hospital (Clare Woodward, Felicity Williams)
- Beckenham Beacon Sexual Health, Kings College Hospital, London (Liz Hamlyn, Lucy Campbell)
- Birmingham Heartlands HIV Service (Steve Taylor, Gerry Gilleran, Satwant Kaur)
- Branston Clinic, Burton-on-Trent (Cathy Ormiston, Laura Wilson-Powell, Kate Saunders)
- Breydon Clinic, iCaSH Norfolk, Great Yarmouth (Meena Gupta, Julia Ball)
- Bristol HIV Service, Southmead Hospital (Mark Gompels, Louise Jennings, Malgorzata Slowinska)
- Brookside Clinic, Aylesbury (Angela Bailey, Sandra Rushwaya)
- Brotherton Wing Clinic, Leeds General Infirmary (Sarah Schoeman, Tadas Mazeika)
- Buryfields Sexual Health Clinic, Surrey Sexual Health Service, Guildford (Shalini Andrews, Laura Noonan)
- Caldecot Centre, Kings College Hospital, London (Liz Hamlyn, Lucy Campbell)
- Cardiff Royal Infirmary (Darren Cousins, Catherine Oliver)
- Chalmers Sexual Health Centre, Edinburgh (Daniel Clutterbuck, Connor Dalby, Amy Shepherd)
- Chesterfield ISHS (Anura Piyadigamage, John Martin)
- Chichester Sexual Health, St Richard’s Hospital (Judith Zhou, Barbara Hayman, Emma Rutland)
- Churchill Hospital, Oxfordshire Sexual Health Service (Paola Cicconi, Charlie Wells)
- Clinic 1a, Addenbrooke’s Hospital, Cambridge (Fiona Wilson)
- Clinic 6, The Oaktree Centre, Huntingdon (Claudia Krause, Su Jenkins)
- Clover Street Clinic, Chatham (Anitha Vidhyadharan, Samantha Harwood)
- Cobridge Community Health Centre, Stoke-on-Trent (Lisa Goodall, Alison Bridgwood, Laura Wilson-Powell)
- Coelho Clinic, Chelmsford (Suzanne Francis, Kirsty Mynard, Mandy Austin)
- Crawley Sexual Health, Crawley Hospital (Judith Zhou, Farai Mukazi, Chloe Hoskins)
- Croydon Sexual Health Centre, Croydon University Hospital, London (Ian Cormack)
- Devon Sexual Health, Barnstaple (Jonathan Shaw, Amanda Smith)
- Devon Sexual Health, Torbay (Nadia Khatib, Julie Walsh)
- Dewsbury Health Centre (Sarah Schoeman, Tadas Mazeika)
- East Kent HIV Service, Folkestone Health Centre (Anitha Vidhyadharan, Brenda Hollier)
- East Sussex Sexual Health, Eastbourne (Martin Jones, Penny Boxall)
- Florence Nightingale Community Hospital, Derby (Ade Apoola, Catherine Gatford)
- Fountains Sexual Health Clinic, Chester (John Evans-Jones, Jennifer Harrison)
- Grahame Hayton Unit, Royal London Hospital, London (Nashaba Matin, Moses Shongwe)
- Greenway Centre, Newham General Hospital, London (Nashaba Matin, Moses Shongwe)
- Harrogate Sexual Health Centre (Ian Fairley)
- Hastings Clinic, Station Plaza, East Sussex Sexual Health (Martin Jones, Zoe Cuthbertson, Penny Boxall)
- Hathersage Centre, Manchester Centre for Sexual Health (Chitra Babu, Denise Donahue)
- Ian Charleson Day Centre, Royal Free Hospital, London (Fiona Burns, Katie Spears, Thomas Fernandez)
- iCaSH Peterborough (Graham McKinnon, Rachael Bridgman)
- Kobler Clinic, Chelsea and Westminster Hospital, London (Ann Sullivan, James Hardie, Mohammed Hassan, Serge Miodragovic)
- Lawson Unit HIV Clinic, University Hospitals Sussex NHS Foundation Trust, Brighton (Amanda Clarke, Lisa Barbour, Carole Cable)
- Luton Sexual Health (Mohanarathi Kawsar, Memory Kakowa)
- Mortimer Market Clinic, London (Richard Gilson, Gosala Gopalakrishnan, Abigail Severn)
- Newington Road Clinic, Ramsgate (Anitha Vidhyadharan, Kate Castro-Sanchez)
- North Manchester General Hospital (Andrew Ustainowski, Fahd Niaz)
- Northampton General Hospital (Sophie Herbert, Helen Reboul)
- Nottingham Sexual Health Service, Nottingham City Hospital (Ashini Fox, Sarah Chadwick)
- Oak Street Clinic, iCaSH Norfolk, Norwich (Nelson David, Megan Khan)
- Open Clinic, Bishton Court, Telford (Andrea Ng, Julia Rogers, Katie Saunders)
- Open Clinic, Sexual Health Services - Shropshire, Shrewsbury (Andrea Ng, Julia Rogers, Katie Saunders)
- Open Clinic, Stafford (Cathy Ormiston, Amandeep Gill, Laura Wilson-Powell, Katie Saunders)
- Portsmouth Sexual Health Service (Alison Blume, Natalie Parker)
- Queen Elizabeth Hospital, Birmingham (Jonathan Ross, Sindiso Masuka)
- Rosehill Clinic, St Helier Hospital, London (Olubanke Davies, Analyn Alipustain, Maheshraj Radhakrishnan)
- Rotherham General Hospital (Nadi Gupta, Nicola Williams)
- Salisbury District Hospital (Helen Iveson)
- Scarborough Sexual Health Centre, The Mulberry Unit (Ian Fairley)
- Sexual Health at Wycombe (Angela Bailey, Sandra Rushwaya)
- Sexual Health Calderdale, Broad Street Plaza, Halifax (Emma Street, Andrew Sealy)
- Sexual Health Clinic, Monkgate Health Centre, York (Ian Fairley, Tom Yucebiyik)
- Sexual Health Dorset, Bournemouth (Elbushra Herieka, Kevin Turner)
- Sexual Health Service, Isle of Wight (Alison Blume, Felicity Young)
- Sexual Health Sheffield, Royal Hallamshire Hospital, Sheffield (Karen Rogstad, Jessica Mcneill, Gareth Stephens)
- SHiP, Derriford Hospital, Plymouth (Zoe Warwick, Angela Robinson, Elaine Freeman)
- Sir Ludwig Guttman Centre, Stratford, London (Nashaba Matin, Moses Shongwe)
- Southend Hospital (Laura Hilton, Donna Stookes)
- Spectrum Community Health, Wakefield (Sarah Schoeman,Tadas Mazeika)
- St Helens Hospital Sexual Health (genitourinary medicine (GUM)) Service (Elizabeth Okecha)
- St Lukes Hospital, Bradford (Nicola Fearnley, Jackie Todd, Sue Kimachia)
- Stevenage Clinic (Ann Sullivan, Sarah Edwards, Mohammed Hassan)
- Summers Unit, Kettering Hospital (Sophie Herbert, Helen Reboul)
- Swindon Sexual Health Department, The Great Western Hospital (Jessica Daniel, Mary-Jane Harding)
- The Centre, Sidwell Street, Exeter (Jonathan Shaw, Abbey Eboigbe, Ashley Hanson)
- The Courtyard Clinic St George’s Hospital, London (Liz Hamlyn, Katie Toler)
- The Florey Sexual Health Services, Royal Berkshire Hospital, Reading (Fabian Chen, Emma Wainwright, Felix Kpodo)
- The Garden Clinic, Upton Hospital, Slough (Nisha Pal, Clare Megson)
- The Gate Clinic, Canterbury (Anitha Vidhyadharan, Matt Waller)
- The James Cook University Hospital, Middlesborough (David Chadwick, Jessica Roberts)
- The Jonathan Mann Clinic, Homerton Hospital, London (Iain Reeves, Tracey Fong)
- The Orwell Clinic, iCaSH Suffolk, Ipswich (Raouf Moussa, Melissa Milsom)
- The Portland Clinic, Huddersfield Royal Infirmary (Emma Street, Mike Ward)
- The Riverside Clinic, Riverside Health Centre, Bath (Lucy Twigger, Charlotte Swift)
- The Royal South Hants Hospital, Southampton (Raj Patel, Jane Whitehead)
- The Starling Clinic, Musgrove Park Hospital, Taunton (Sathish Thomas William, Jane Holder)
- The Trafalgar Clinic, Queen Elizabeth Hospital, Greenwich, London (Stephen Kegg, Rosa Harrington, Allison Mascagni, Claudia Adade)
- The Wolverton Centre for Sexual Health, Kingston Hospital, London (Lewis Haddow, Jessica Osorio)
- Twickenham House, West Middlesex Hospital, London (Ann Sullivan, Marie-Louise Svensson)
- Vancouver House, iCaSH Norfolk, King’s Lynn (Sandra Underwood, Helen Pollitt)
- Vicarage Lane Clinic, Ashford (Anitha Vidhyadharan, Brenda Hollier)
- Watford Clinic (Ann Sullivan, Samantha Hill)
- Weymouth Community Hospital, Sexual Health Dorset, Weymouth (Sara Scofield, Jenny Murira)
- Wharfside Clinic, St Mary’s Hospital, London (Nicola Mackie, Sophia Taylor, Romina Tajik)
- Withington Community Hospital, Manchester (Orla McQuillan, Denise Donahue)
- Worthing Sexual Health (Judith Zhou, Rebecca Murdock, Elaine Banks)
